# Human cells are permissive for the productive infection of porcine circovirus type 2 *in vitro*

**DOI:** 10.1038/s41598-019-42210-0

**Published:** 2019-04-04

**Authors:** Xiaohui Liu, Ting Ouyang, Hongsheng Ouyang, Xiaohua Liu, Guyu Niu, Wang Huo, Weihong Yin, Daxin Pang, Linzhu Ren

**Affiliations:** 1 Jilin Provincial Key Laboratory of Animal Embryo Engineering, College of Animal Sciences, Jilin University, 5333 Xi’an Road, Changchun, 130062 China; 2 College of Life Sciences, Shandong Normal University, Jinan, 250014 China

## Abstract

Porcine circovirus 2 (PCV2) is the main pathogen of porcine circovirus diseases and porcine circovirus-associated diseases, which are widespread in swine-producing countries. However, there is controversy regarding the susceptibility of human cells to PCV2 infection. In this study, human cell lines were infected with PCV2 and blind passaged several times. PCV2 entered and replicated in human cells, and infectious virions were generated, indicating that human cell lines were permissive to PCV2 replication. Furthermore, PCV2 replication in human cell lines was enhanced by D-glucosamine or concanavalin A (ConA). However, the infection efficiency of PCV2 was lower in human cells than in PK-15 cells, suggesting that PCV2 infection was limited in human cells. Our study reveals that human cells are permissive for the productive infection of porcine circovirus type 2 *in vitro*.

## Introduction

Porcine circovirus (PCV) belongs to the genus *Circovirus* in the family *Circoviridae* and contains a single-stranded 1.7-kb circular DNA^1–4^. There are three types of PCV: porcine circovirus type 1 (PCV1), PCV2, and PCV3. PCV1 is non-pathogenic and considered a contaminant of the porcine kidney cell line (PK-15)^4,5^. Recently, some groups reported that commercial human rotavirus vaccines and porcine-derived pepsin products were contaminated with PCV1 and PCV2 DNA^5–8^. Unexpectedly, it was found that PCV1 can infect human 293 T, HeLa, and Chang liver cells without causing any visible changes^9^. Infectious PCV1 was detected in the lysate of infected human hepatocellular carcinoma cells and was serially passaged in the cells^5^. Another group found that PCV1 infection caused ultrastructural alterations of infected human cells^10^. As the genomic sequence of PCV2 shows 80% overall nucleotide sequence identity with that of PCV1^11^, it is easy to assume that PCV2 may infect human cells. Nevertheless, to date, there is controversy regarding the susceptibility of human cells to PCV2 infection.

PCV2 was first confirmed in 1982 and subsequently identified in pigs in the USA, France, Japan, Korea, China, and other countries^1,4,12–15^. PCV2 is the main pathogen of porcine circovirus diseases and porcine circovirus-associated diseases (PCVD/PCVAD), which are widespread in swine-producing countries^1,4,16,17^. PCV2 DNA was amplified from PCV2-transfected 293 T, HeLa, Hep2, RH, and Chang liver cells, and the expression of viral antigen was observed in all cells^9^. A CPE was observed in PCV2-transfected cells 3 days post-infection (dpi); the cells were altered in morphology from stretched to round, and the number of dead cells and cell debris was increased in the supernatant^9^. However, the PCV2 signal was lost after 2 weeks, and viral particles were not produced^9^. Investigations performed by other groups showed no evidence for the existence of PCV2-specific antibodies in the sera of PCV2-exposed persons, indicating that PCV2 infection in human cells was non-productive^18–20^. Surprisingly, 235 (28.5%) samples of 826 stool swabs collected from 102 children who received a live rotavirus vaccine were positive for PCV-2 DNA^21^. Therefore, it is urgent to determine whether human cells are permissive for PCV2 infection and replication.

## Results

### Human cell lines are susceptible to PCV2 infection

**Figure 1:**
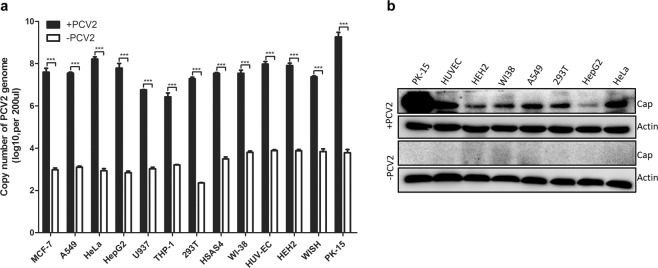
Human cell lines are susceptible to PCV2 infection. Cancerous human cell lines (MCF-7, A549, HeLa, HepG2, U937, THP-1) and normal human cell lines (293 T, WI-38, HUVEC, WISH, HSAS4, HEH2) were infected with PCV2 at an MOI of 5 for 72 h. The viral DNA was quantified by SYBR Green quantitative real-time PCR, and viral proteins were detected by Western blot. Cells that were not infected with PCV2 were used as control cells. (**a**) SYBR Green quantitative real-time PCR. (**b**) Western blot. Western blot was performed using the porcine circovirus type 2/PCV2 Capsid antibody or mouse Beta actin Antibody (1:2000) as the primary antibody and HRP-conjugated goat anti-rabbit IgG or HRP-conjugated goat anti-mouse IgG as the secondary antibody. Unprocessed original scans of the Western blots can be found in Supplementary Fig. S1.

To investigate whether human cells are susceptible to PCV2 infection, twelve human cell lines, including six cancer cell lines and six normal cell lines, were infected with PCV2 at a multiplicity of infection (MOI) of 5 for 72 h. PCV2 genomic DNA was detected in all the human cells as well as the PK-15 cells (Fig. 1a). The PCV2 DNA copy numbers were approximately 10^6.5^ to 10^8.5^ copies/200 μL in the human cell lines examined in this study. Furthermore, Western blotting was performed to confirm viral expression. The viral Cap protein was detected in human cells as well as PK-15 cells infected with PCV2, while no protein was observed in non-infected cells (Fig. 1b).

**Figure 2:**
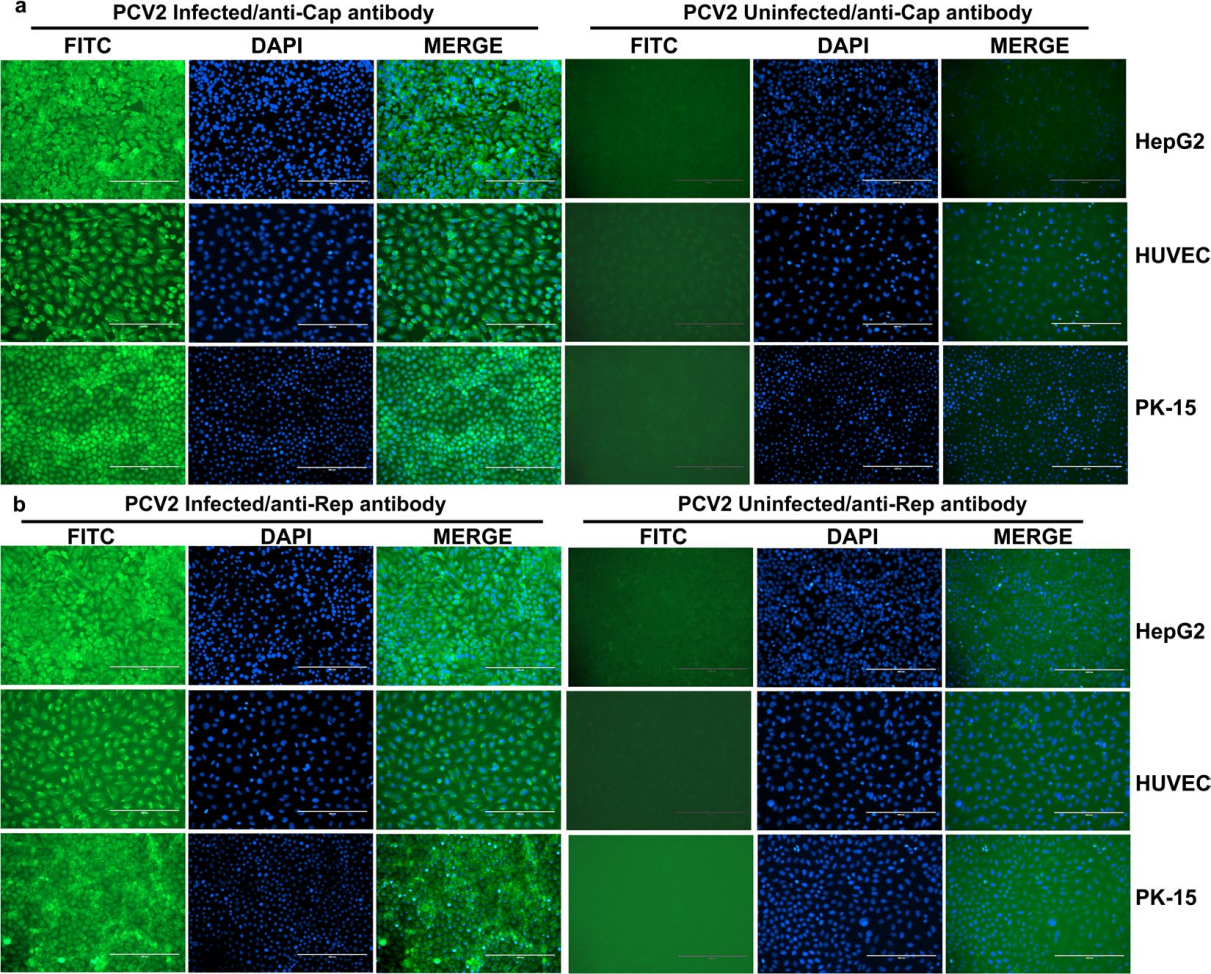
Immunostaining of cells infected with the CC1 strain of PCV2 72 hpi. Cells were infected with PCV2 at an MOI of 5 for 72 h and stained with rabbit anti-Cap antibody (1:100) (**a**) or rabbit anti-Rep antibody (1:100) (**b**), followed by staining with FITC-labelled goat anti-rabbit IgG (H + L) (green, 1:1000). DAPI (blue) was used to stain nuclei. Cells were visualized using Eclipse TE2000-V (Nikon).

Cap and Rep, which are the main proteins of PCV2, are a viral structural protein and a viral DNA replication-associated protein, respectively^4^. To further confirm infection of human cells with PCV2, viral growth was analysed by indirect immunofluorescence assay (IFA), using either rabbit anti-Cap antibody (Fig. 2a) or rabbit anti-Rep antibody (Fig. 2b) as the primary antibody, both of which were previously prepared in our lab^22,23^. The subcellular distributions of both Cap and Rep were observed 72 h after PCV2 infection. The fluorescence was significantly greater in PCV2-infected human cells and PK-15 cells than that of control cells (mock-infected cells), indicating that both the Cap and Rep proteins of PCV2 were expressed in human cells as well as PK-15 cells.

Taken together, these results demonstrated that the human cell lines used in the present study are susceptible to PCV2 infection.

### Human cell lines support PCV2 replication, and progeny viruses are infectious

**Figure 3:**
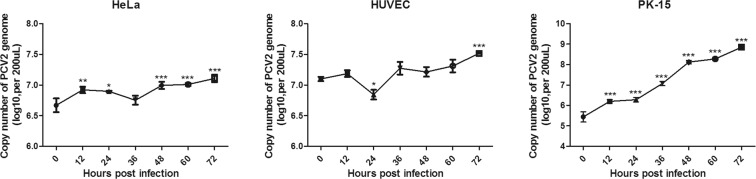
One-step growth curve of PCV2 in different cells. HeLa cells, HUVECs and PK-15 cells were infected with PCV2 strain CC1 at an MOI of 5 for 72 h at 37 °C in a 5% CO_2_ atmosphere for 1 h. Thereafter, the infected cells were washed with PBS three times to remove potential free viruses that had not entered the cells, and then the PK-15 cells and human cells were cultured in fresh culture medium containing 2% FBS or 5% FBS, respectively (This time point was considered as 0 hpi.). Samples were collected at 0, 12, 24, 36, 48, 60 and 72 hpi. The viral DNA was quantified by SYBR Green quantitative real-time PCR. **p < 0·01; ***p < 0·001; ns, no significance.

To evaluate whether PCV2 can replicate in human cells, a one-step growth curve was performed in HeLa cells, HUVECs and PK-15 cells. Cells inoculated with PCV2 at an MOI of 5 were collected at intervals of 12 h. The viral DNA was quantified by SYBR Green quantitative real-time PCR. The amount of viral DNA gradually increased from 12 to 72 h (Fig. 3). PCV2 propagated more efficiently in PK-15 and HeLa cells and had a significantly higher copy number of viral DNA by 72 h post-infection (hpi) than in HUVECs, whereas the amount of viral DNA was lower at 24 hpi and increased from 36 to 72 hpi in HUVECs. Among the two human cell lines, viruses grown in HUVECs exhibited better growth than those grown in HeLa cells, as they exhibited viral DNA copy numbers of approximately 3.3 × 10^7^ and 1.3 × 10^7^ copies/200 μL by 72 hpi, respectively. Interestingly, although the cells were inoculated with similar copy numbers of PCV2 genomic DNA followed by attachment for one hour, PCV2 genomic DNA was detected at approximately 4.8 × 10^6^ and 1.6 × 10^7^ copies/200 μL in HeLa and HUVECs at 0 hpi, respectively, which were statistically higher than that in PK-15 cells (3.0 × 10^5^ copies/200 μL).

**Figure 4:**
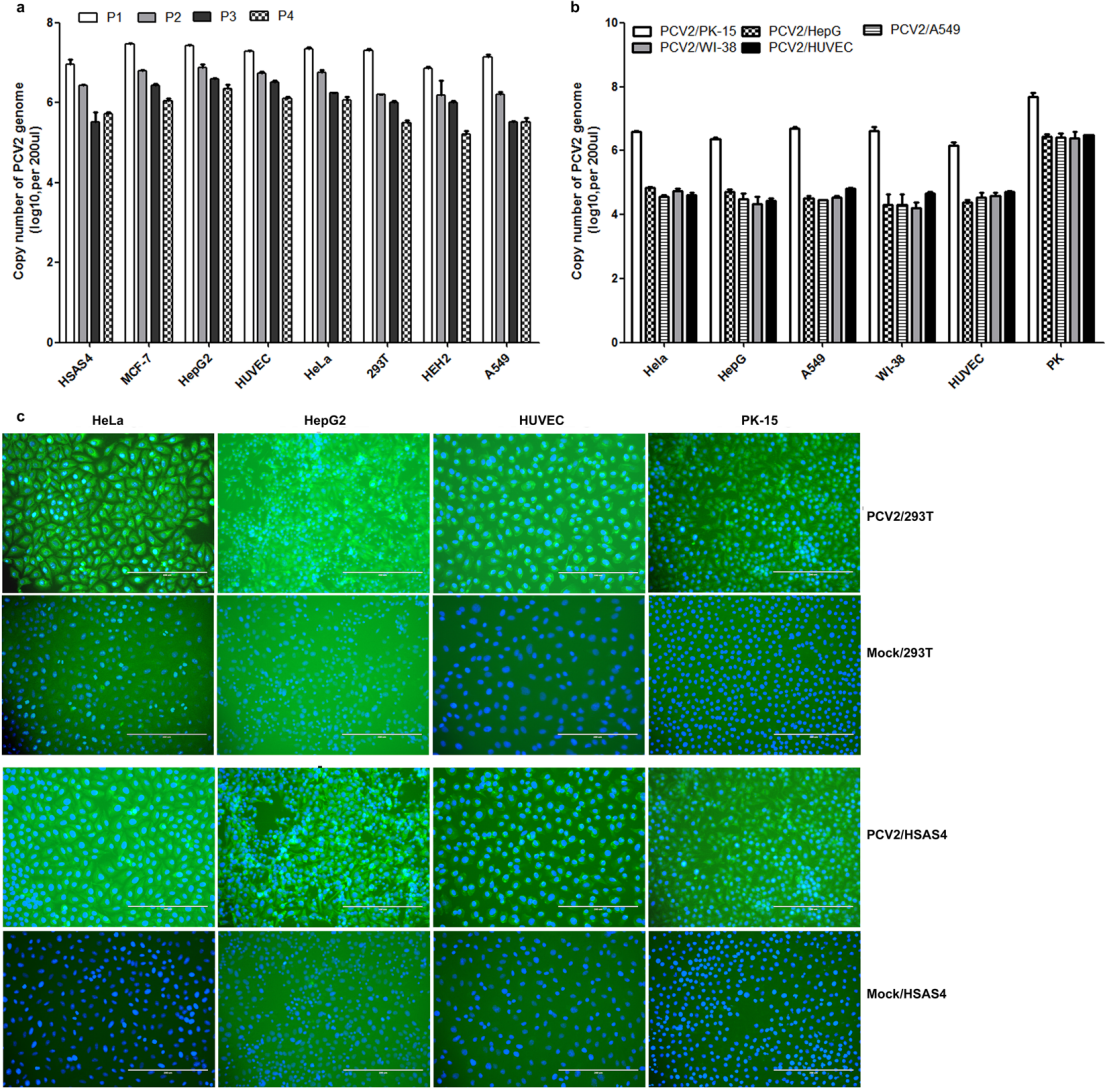
Progeny viral particles are infectious in human cells. (**a**) PCV2 can replicate in serially passaged human cell lines. Cells were infected with PCV2 for 72 h, and the resultant cells were blind passaged four times, followed by SYBR Green quantitative real-time PCR. P1, the first passage; P2, the second passage; P3, the third passage; and P4, the fourth passage. (**b**) PCV2 collected from one cell line can infect other cell lines. Supernatants were collected from PCV2-infected cells (PK-15, HepG2, A549, or WI-38 cells or HUVECs) after three cycles of freezing and thawing. The supernatants were used to infect HeLa, HepG2, A549, WI-38, PK-15 cells and HUVECs. At 72 hpi, SYBR Green quantitative real-time PCR was performed to detect the PCV2 copy number in the cells. PCV2/PK-15, PCV2 collected from PK-15 cells; PCV2/HepG2, PCV2 collected from HepG2 cells; PCV2/A549, PCV2 collected from A549 cells; PCV2/WI-38, PCV2 collected from WI-38 cells; PCV2/HUVEC, PCV2 collected from HUVECs. (**c**) Indirect immunofluorescence assay (IFA). Supernatants were collected from PCV2-infected cells (293 T or HSAS4) after three cycles of freezing and thawing. The supernatants were used to infect HeLa, HepG2, PK-15 cells and HUVECs. At 72 hpi, the cells were stained with rabbit anti-Cap antibody (1:100), followed by staining with FITC-labelled goat anti-rabbit IgG (H + L) (green, Beyotime, 1:1000). DAPI (blue) was used to stain nuclei. Cells were visualized using Eclipse TE2000-V (Nikon). PCV2/293 T, PCV2 collected from 293 T cells; PCV2/HSAS4, PCV2 collected from HSAS4 cells. Supernatants collected from non-infected 293 T or HSAS4 cells were used as negative controls (mock).

To further investigate the growth characteristics of PCV2 in human cells, PCV2-infected cells were blind passaged four times, and PCV2 DNA copy numbers were analysed using real-time PCR. PCV2 proliferated consistently in four passages of human cells as well as PK-15 cells (Fig. 4a), suggesting that the virus can replicate in human cells and that the progeny viruses were infectious. To confirm this result, the PCV2-infected cells were lysed by three cycles of freezing and thawing, and the supernatants were transferred to other virus-free cells, followed by detection of the viral DNA at 72 hpi. Real-time PCR demonstrated that the progeny virions from one human cell and PK-15 cell can also replicate in virus-free PK-15 cells and other virus-free human cells (Fig. 4b). Moreover, supernatants collected from PCV2-infected or non-infected 293 T or HSAS4 cells were incubated with different human cells for 72 h, followed by IFA analysis. The results of IFA showed that the fluorescence was significantly increased in the human cells incubated with supernatants collected from PCV2-infected 293 T or HSAS4 (Fig. 4c) cells compared with that in non-infected cells.

**Figure 5:**
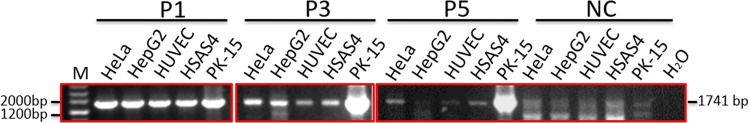
PCR detection of the full-length PCV2 genome. Cells were infected with PCV2 for 72 h, and the resultant viruses were blind passaged five times, followed by the identification of PCV2 by PCR according to the protocol described by Gilliland *et al*.^7^. The expected size of the PCR product from PCV2 was 1741 bp. H_2_O and DNA from uninfected cells were used as negative controls. M, DNA marker. P1, the first passage; P3, the third passage; and P5, the fifth passage. NC, negative control. Unprocessed original scans of the gel can be found in Supplementary Fig. S2.

Furthermore, to confirm the presence of PCV2 genomic DNA, conventional PCR using primers described by Gilliland *et al*.^7^ was performed to detect PCV2 DNA in the cell supernatants from passages 1, 3 and 5. A PCV2-specific product of the expected size, a 1741 bp product of the 1766 bp full-length PCV2 DNA genome, was amplified from all the cell supernatants from passages 1, 3 and 5 (Fig. 5). However, we did not detect PCV2-specific PCR products in the negative control. These results confirmed the presence of near full-length genomic PCV2 DNA in PCV2-infected cells, indicating that the viral DNA was replicated in human cells.

**Figure 6:**
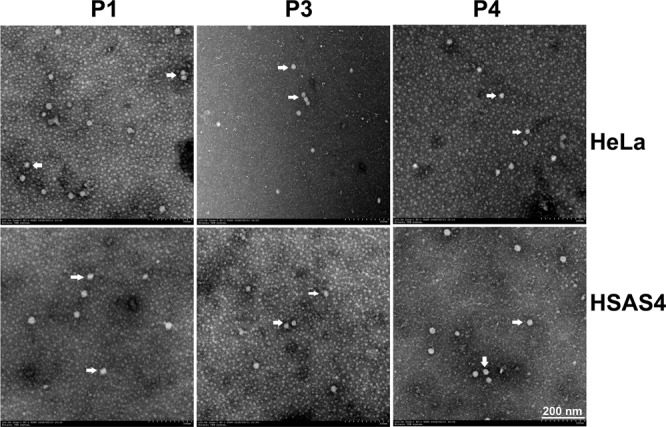
Transmission electron microscopy (TEM) ultrastructural analysis. PCV2 particles 20 nm in diameter were observed by TEM. Scale bar indicates 200 nm. P1, the first passage; P3, the third passage; P4, the fourth passage.

Moreover, electron microscopy was performed to visualize virions in human cells. Two human cell lines, HeLa and HSAS4, were randomly selected and infected with PCV2, and viruses were blind passaged in the respective cells. Cultures were then prepared and examined by transmission electron microscopy. Spherical virions with a diameter of 20 nm were observed in cells from each passage of the PCV2-infected human cells (Fig. 6).

Together, these results demonstrated that human cells are permissive for PCV2 infection and supported the *in vitro* replication of PCV2.

### PCV2 infection in human cell lines is enhanced by D-glucosamine or concanavalin A (ConA)

**Figure 7:**
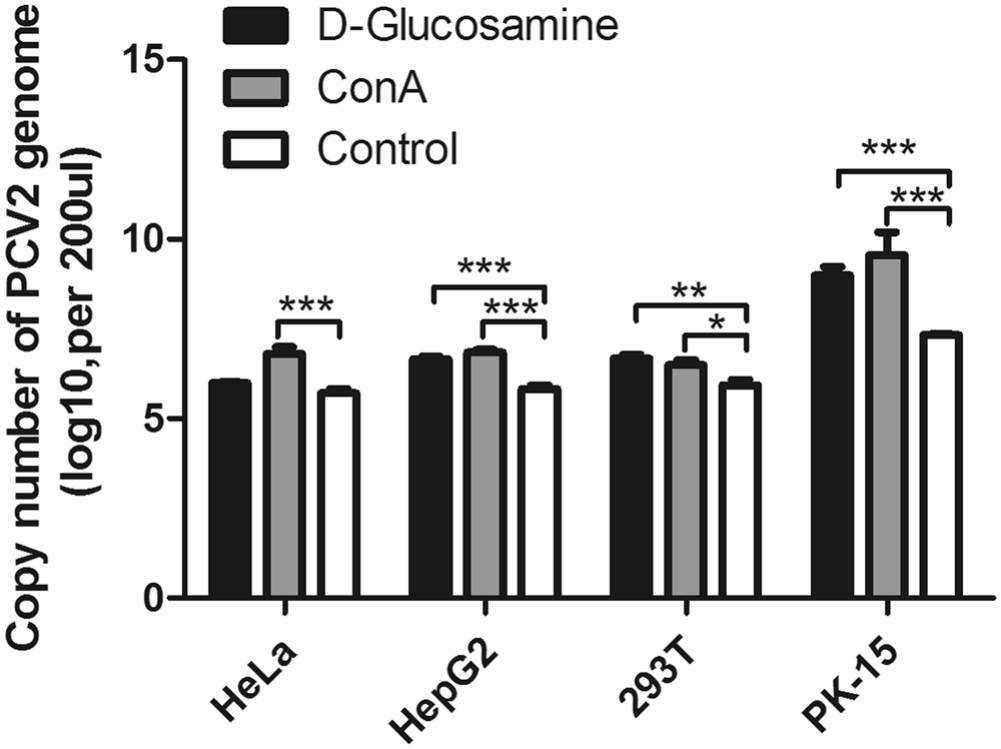
PCV2 infection in human cells is enhanced by D-glucosamine or ConA. Cells were inoculated with PCV2 for 1 h, washed with PBS three times and maintained in fresh culture medium containing 5 μg/mL ConA or 300 mM D-glucosamine and 2% FBS in the case of PK-15 cells or 5% FBS in the case of human cells. Control cells were inoculated with PCV2 for 1 h and maintained in fresh culture medium containing 2% FBS in the case of PK-15 cells or 5% FBS in the case of human cells. At 72 hpi, cells were lysed by freezing and thawing three times, and the viral DNA was quantified by SYBR Green quantitative real-time PCR. **p < 0·01; ***p < 0·001; ns, no significance.

Previously, we and other groups found that IL-2, ConA, and D-glucosamine significantly increased PCV2 yield^24–26^. Therefore, we assumed that treatment with ConA and D-glucosamine would enhance PCV2 infection in human cells. To confirm our hypothesis, three human cell lines and PK-15 cells were infected with PCV2 and treated with ConA or D-glucosamine. The amount of viral genomic DNA was significantly enhanced in PCV2-infected HeLa, HepG2, 293 T and PK-15 cells compared with that in untreated cells (Fig. 7). These results indicated that PCV2 infection in human cell lines can be enhanced by ConA or D-glucosamine, which is consistent with results obtained in PK-15 cells.

## Discussion

In the present study, we reported that human cells were permissive to productive PCV2 infection and that they supported viral replication *in vitro*. We infected twelve human cell lines with PCV2 and found that all human cells in the present study were susceptible to PCV2 infection, indicating that PCV2 has a broad cell tropism and can infect many types of human cells, including monocytes, epithelial cells and fibroblasts. However, the PCV2 DNA copy numbers were more than 10^7^ copies/200 μL in most of the human cell lines, except for U937 and THP-1 cells (Fig. 1). It is possible that U937 and THP-1 cells are suspension cells, which may affect viral infection and alter it compared with that in adherent cells. In addition, both U937 and THP-1 cells are monocytes, which may interfere or suppress PCV2 infection. Further research is in progress to determine the reason for this difference.

PCV2 is an extremely slow-growing virus, and PCV2 infection and replication in PK-15 cells yielded very low viral titers^11,24^. Viral antigens were only observed in a few cells at 18 hpi, and cell-free progeny viruses began to appear at approximately 30 hpi^27^. Previously, we and other groups found that IL-2, ConA, and D-glucosamine significantly increased PCV2 yield^24–26^. However, the infection efficiency of PCV2 was lower in all human cell lines than that in PK-15 cells (Fig. 4a,b), even though the human cells were treated with ConA or D-glucosamine, which significantly enhanced PCV2 infection in PK-15 cells (Fig. 7). One possible reason is that human cells are not the natural host cells of PCV2 and cannot provide a suitable cellular environment for the sustainable replication of PCV2. Moreover, although the cells were inoculated with similar copy numbers of PCV2 genomic DNA followed by attachment for one hour, PCV2 genomic DNA was detected at a concentration of approximately 4.8 × 10^6^ and 1.6 × 10^7^ copies/200 μL in HeLa and HUVECs at 0 hpi, respectively, and these concentrations were statistically higher than that in PK-15 cells (3.0 × 10^5^ copies/200 μL) (Fig. 3), indicating that PCV2 can attach more easily to human cells than PK-15 cells. Analysis of the interactions between PCV2 and human cells is ongoing. Additionally, *in vitro* and *in vivo* experiments are very different, and animal experiments (e.g., primates) will be used to confirm these results in our future research.

PCV2 has immunosuppressive properties^17^, which may increase susceptibility to opportunistic infection. Moreover, PCV2 infection can downregulate immune cell functions during recall antigen responses and induce IL-10 secretion by monocytic cells, leading to the effective repression of IL-12 in porcine PBMCs^28^. Therefore, whether PCV2 infection in human cells affects the cellular immune response needs to be elucidated. Furthermore, it was reported that singular PCV2 infection in pigs rarely results in clinical disease, while PCVD/PCVAD is often enhanced in severity and prolonged in duration with concurrent viral or bacterial infections^4,29^. Thus, whether co-infection of PCV2 with other pathogens occurs in human cells needs to be investigated.

## Conclusions

In conclusion, the present study demonstrated that PCV2 is capable of infecting human cells *in vitro*. PCV2 can enter and replicate in human cells, resulting in infectious virions. It is noteworthy that the infection efficiency of PCV2 was lower in human cell lines than in PK-15 cells, indicating that PCV2 infection was limited in human cells under the current experimental conditions. Therefore, whether PCV2 is a zoonotic pathogen needs to be further investigated.

## Methods

### Virus and cells

PCV2 strain CC1 (GenBank accession no. JQ955679) was isolated and stored in our lab^30^.

Six cancerous human cell lines (MCF-7, A549, HeLa, HepG2, U937, THP-1) and six normal human cell lines (293 T, WI-38, HUVEC, WISH, HSAS4, HEH2) were purchased from Shanghai Tong Wei Biological Technology Co., Ltd. (China) and used for PCV2 infection. PCV-free PK-15 cells were stored in our lab and used as a control. For information on the cells used in this study, please see Supplementary Table S1.

### Virus infection

Cells were cultured in Dulbecco’s modified Eagle’s medium (DMEM, Gibco, USA) supplemented with 10% foetal bovine serum (FBS, Gibco, USA) at 37 °C in a 5% CO_2_ atmosphere. At 30–50% confluency, cells were infected with PCV2 strain CC1 at 37 °C in a 5% CO_2_ atmosphere for 1 h. Thereafter, the infected cells were washed with PBS three times to remove potential free viruses that had not entered the cells, and then the PK-15 and human cells were cultured in fresh culture medium containing 2% FBS or 5% FBS, respectively. Seventy-two hours post-infection (hpi), cells were lysed by freezing and thawing three times while shaking the half-frozen medium and stored at −80 °C. The viral DNA was quantified by SYBR Green quantitative real-time PCR, and the viral protein was detected by Western blot.

To determine whether human cell lines support productive PCV2, serial passages were performed in PCV2-infected human cells and PK-15 cells. Briefly, PCV2-infected cells were washed with PBS three times and then trypsinized by elution with 0.25% trypsin. The resuspended cells were maintained 1:1 in fresh culture medium containing 4% FBS in the case of PK-15 cells or 10% FBS in the case of human cells for 24 h, and the resultant cells were blind passaged five times. The viral DNA at each passage was quantified by SYBR Green quantitative real-time PCR.

To evaluate whether ConA and D-glucosamine enhanced PCV2 infection in human cells, PCV2-infected cells were cultured in fresh culture medium containing 5 μg/mL ConA (Sigma, USA) or 300 mM D-glucosamine (Sigma, USA) and 2% FBS in the case of PK-15 cells or 5% FBS in the case of human cells. Control cells were inoculated with PCV2 for 1 h and maintained in fresh culture medium containing 2% FBS in the case of PK-15 cells or 5% FBS in the case of human cells. Seventy-two hours post-infection, cells were lysed by freezing and thawing three times, and the viral DNA was quantified by SYBR Green quantitative real-time PCR.

### Polymerase chain reaction (PCR) and quantitative real-time PCR

PCV2 DNA was extracted from infected cells using a TIANamp Virus DNA/RNA Kit (Tiangen Biotech, Beijing, China) according to the manufacturer’s protocol. The genomic DNA was dissolved in 50 μL of DNase-free ddH_2_O and stored at −20 °C. PCR was performed to detect the full-length PCV2 genome according to the protocol described by Gilliland *et al*.^7^. SYBR Green quantitative real-time PCR was performed according to the protocol described by Yang *et al*.^24^ using the Bio-Rad IQ^TM^ Multicolor Real-Time PCR Detection System and the Luna^®^ Universal qPCR Master Mix (New England Biolabs, USA). The primers used in this study are listed in Supplemental Table 1 (Table S1). The experiments were repeated at least three times.

### Western blot (WB)

Western blot was performed according to the protocol described by Yang *et al*.^31^. Briefly, whole-cell lysates were separated by SDS-PAGE, electro-transferred to PVDF membranes (Millipore), blocked for 1 h with 5% skim milk in TBS-T buffer (20 mM Tris-HCl [pH 7.4], 150 mM NaCl, and 0.1% Tween-20), and then incubated with the porcine circovirus type 2/PCV2 Capsid antibody (1:500, GeneTex, Texas, USA) or mouse Beta actin Antibody (1:2000, Proteintech Group, USA) at 4 °C overnight. Subsequently, the PVDF membranes were washed with 5% skim milk in TBS-T buffer three times, followed by blotting for 1.5 h with HRP-conjugated goat anti-rabbit IgG or HRP-conjugated goat anti-mouse IgG (1:5000, Beyotime, Jiangsu, China). The immunoreactive bands were visualized using the BeyoECL Plus Western blot detection system (Beyotime, Jiangsu, China) according to the manufacturer’s instructions. The experiments were repeated at least three times.

### Indirect immunofluorescence assaNy (IFA)

The IFA assay was performed as described by Yang *et al*.^24^. Briefly, cells in 24-well plates were infected with PCV2 for 72 h and fixed with 80% ice-cold acetone at −20 °C overnight. The cells were then washed three times with PBS-Tween (PBS-T) and incubated with rabbit anti-Cap antibody or rabbit anti-Rep antibody (1:100) at 4 °C for 12 h. After an additional five washes with PBS-T, the cells were incubated with FITC-labelled goat anti-rabbit IgG (H + L) (1:1000, Beyotime, Jiangsu, China) at 37 °C for 30 min. After an additional five PBS-T washes, the cells were incubated with DAPI (Beyotime, Jiangsu, China) at 37 °C for 8 min, followed by washing with PBS-T three times. Thereafter, the cells were examined using an Eclipse TE2000-V (Nikon). Antibodies against PCV2 proteins, rabbit anti-Cap antibody or rabbit anti-Rep antibody, were previously prepared and stored in our lab^22,23^.

### One-step growth curve

HeLa cells, HUVECs and PK-15 cells were infected with PCV2 at an MOI of 5 at 37 °C in a 5% CO_2_ atmosphere for 1 h. Thereafter, the infected cells were washed with PBS three times to remove potential free viruses that had not entered the cells, and then the PK-15 cells and human cells were cultured in fresh culture medium containing 2% FBS or 5% FBS, respectively (This time point was considered as 0 hpi.). Samples were collected at 0, 12, 24, 36, 48, 60 and 72 hpi. The viral DNA at different time points was quantified by SYBR Green quantitative real-time PCR.

### Electron microscopy (EM) analyses

Cells were infected with PCV2 for 48 h and blind passaged four times. PCV2-infected cells at each passage were lysed by freezing and thawing three times while shaking the half-frozen medium. Cell fractions containing viral particles were collected and examined by negative staining transmission electron microscopy (Hitachi TEM system).

### Statistical analysis

Statistical analysis was performed using GraphPad Prism software, version 5 (GraphPad Software, San Diego, CA). For each separate set of assays, at least 3 independent experiments were evaluated. The results are expressed as the mean ± standard deviation (SD). The results were considered statistically significant at p < 0.05.

## Supplementary information

Supplementary informations

## Data Availability

The data used and/or analysed in the current study are available from the corresponding author on reasonable request.
