# Supplementary material for: Human cells are permissive for the productive infection of porcine circovirus type 2 in vitro
Source: Sci Rep. 2019 Apr 4;9:5638. doi: 10.1038/s41598-019-42210-0 (PMC6449348; doi:10.1038/s41598-019-42210-0)
Supplement: Supplementary file 1 — Supplementary informations [file 41598_2019_42210_MOESM1_ESM.pdf]

Human cells are permissive for productive infection of porcine circovirus type 2 *in vitro*

Xiaohui Liu[1*], Ting Ouyang[1*], Hongsheng Ouyang[1*], Xiaohua Liu[1], Guyu Niu[1], Wang Huo[1], Weihong Yin[1], Daxin Pang[1], Linzhu Ren[1, 2#]

[1] Jilin Provincial Key Laboratory of Animal Embryo Engineering, College of Animal Sciences, Jilin University, 5333 Xi'an Road, Changchun 130062, China
[2] College of Life Sciences, Shandong Normal University, Jinan 250014, China

[#]**Correspondence**: Linzhu Ren, Jilin Provincial Key Laboratory of Animal Embryo Engineering, College of Animal Sciences, Jilin University, 5333 Xi'an Road, Changchun, Jilin 130062, China; E-mail: renlz@jlu.edu.cn

This file includes:

Table S1 to S2

Figs. S1 to S2

Table S1 Information of cells used in this study.

Cell	Organism	Company	Catalogue No.	Tissue	Morphology	Culture Properties	Disease
MCF-7	Human	ATCC	ATCC HTB-22	mammary gland, breast	epithelial	adheren	adenocarcinoma
A549	Human	ATCC	ATCC CRM-CCL-185	lung	epithelial	adherent	carcinoma
Hela	Human	ATCC	ATCC CCL-2	cervix	epithelial	adherent	adenocarcinoma
Hep G2	Human	ATCC	ATCC HB-8065	liver	epithelial	adherent	hepatocellular carcinoma
U937	Human	ATCC	ATCC CRL-1593	pleura/pleural effusion, lymphocyte, Myeloid	monocyte	suspension	histiocytic lymphoma
THP-1	Human	ATCC	ATCC TIB-202	peripheral blood	monocyte	suspension	acute monocytic leukemia
HUVEC	Human	ATCC	ATCC CRL-1730	umbilical vein/vascular endothelium	endothelial	adherent	normal
WISH	Human	ATCC	ATCC CCL-25	amnioncells	epithelial	adherent	normal
293T	Human	ATCC	ATCC CRL-3216	embryonic kidney	epithelial	adherent	normal
WI-38	Human	ATCC	ATCC CCL-75	lung	fibroblast	adherent	normal
HSAS4	Human	Kuming Cell Bank, CAS	KCB 200537	skin, foreskin	fibroblast	adherent	normal
HEH2	Human	Kuming Cell Bank, CAS	KCB200505EH	embryonic cardiac-muscle	fibroblast	adherent	normal
PK-15	Sus scrofa	ATCC	ATCC CCL-33	kidney	epithelial	adherent	normal

Table S2 Primers used in this study.

Primers	Sequence	Product size (bp)	References
PCV2fullfw	5-CACTTCGTAATGGTTTTTATTTTTCA-3	1741	Gilliland *et al.* [1]
PCV2fullrev	5-TATGGAAATTCAGGGCATGG-3		
P1	5′-TGTAGTATTCAAAGGGCACAGAGC-3′	130	Yang *et al.* [2]
P2	5′-CGGATATACTATCAAGCGAACCAC-3′		

Fig S-1

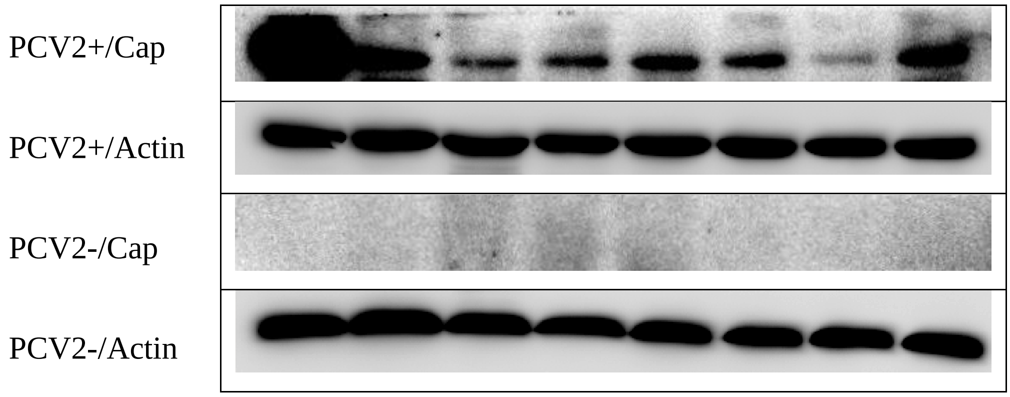

Fig S-1: Original Western images used for preparing Figure 1a.

Fig S-2

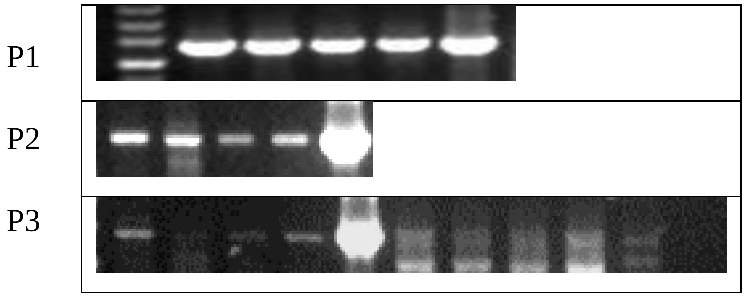

Fig S-2: Original Western images used for preparing Figure 5.

References

1. Gilliland SM*, et al.* Investigation of porcine circovirus contamination in human vaccines. *Biologicals* **40**, 270-277 (2012).

2. Yang X, Chen F, Cao Y, Pang D, Ouyang H, Ren L. Comparative analysis of different methods to enhance porcine circovirus 2 replication. *J Virol Methods* **187**, 368-371 (2013).
